# Telemedicine during the COVID-19 pandemic in Germany: Results from three nationally representative surveys on use, attitudes and barriers among adults affected by depression

**DOI:** 10.1016/j.invent.2023.100622

**Published:** 2023-04-19

**Authors:** S. von der Groeben, A. Czaplicki, U. Hegerl, H. Reich

**Affiliations:** a Goethe University Frankfurt, University Hospital, Depression Research Centre of the German Depression Foundation, Department of Psychiatry, Psychosomatic Medicine and Psychotherapy, Germany; b German Depression Foundation, Leipzig, Germany; c Johann Christian Senckenberg Distinguished Professorship, Department of Psychiatry, Psychosomatic Medicine and Psychotherapy, University Hospital, Goethe University, Frankfurt am Main, Germany

**Keywords:** Telemedicine, E-health, Depression, COVID-19, Representative survey, Quasi-longitudinal study

## Abstract

**Introduction:**

The COVID-19 pandemic has necessitated a reduction in face-to-face consultations, resulting in significant limitations in healthcare for individuals with depression. To ensure safe and adequate care, e-health services, such as telemedicine, gained a more prominent role. Governments have eased restrictions on the use of telemedicine, enabling healthcare professionals to increasingly offer video and telephone consultations.

**Objective:**

This study examines, 1) possible changes over the course of the pandemic in reported use of video and telephone consultations and intended future use of video consultations with healthcare professionals among adults with diagnosed depression; 2) their attitudes towards video and telephone consultations and perceived barriers towards using e-health after prolonged time of the pandemic; and 3) differences in results between subgroups based on sociodemographic and clinical characteristics.

**Methods:**

Three population-representative online surveys were conducted in Germany at different timepoints (t) during the COVID-19 pandemic. Respondents aged 18–69 years with a professionally diagnosed depression were included in the present analyses (t1: June/July 2020 with n = 1094; t2: February 2021 with n = 1038; t3: September 2021 with n = 1255).

**Results:**

The overall proportion of adults with depression who used video or telephone consultations did not change significantly in the time surveyed (t1: 16.51 %, n = 179; t2: 20.23 %, n = 210; t3: 18.47 %, n = 230). However, among users, reported use of video consultations with a psychotherapist increased significantly from t1 (34.83 %, n = 62) to t3 (44.98 %, n = 102, *p* = .023). Intended future use of VC for healthcare varied depending on the purpose of the consultation. Significant differences over time were only found for the purpose of using VC to discuss clinical findings, laboratory results and diagnostic analyses with a doctor, with higher intentions reported at t2 during lockdown in Germany. At t3, the majority of adults with depression felt that video and telephone consultations were too impersonal and considered them more as a helpful support rather than an alternative to face-to-face psychotherapy. Key barriers to using e-health were found within the societal context and the lacking support from significant others for using e-health, while knowledge and skills represented facilitators for using e-health.

**Conclusion:**

Despite ambivalent attitudes towards video and telephone consultations among adults with depression, reported use of video consultations with a psychotherapist increased during the COVID-19 pandemic.

## Highlights

• The use of video consultations with a psychotherapist increased significantly during the COVID-19 pandemic
• Telemedicine was considered more as a helpful support than an alternative to face-to-face consultations or pharmacotherapy
• Key barriers to e-health were related to societal factors: Both institutional and private support were deemed insufficient.

## 1 Introduction

Depression is a severe and potentially life-threatening mental illness that affects >300 million people worldwide (Salud, 2017), making it a leading cause for the global burden of disease (Abbafati et al., 2020). However, >80 % of people suffering from depression do not receive adequate treatment in line with national guidelines (DGPPN, BÄK, KBV, AWMF, 2015; Thornicroft et al., 2017) Barriers to accessing treatment include lack of available services, difficulties in reaching nearby healthcare providers, long waiting lists, and social stigmatization (Kohn et al., 2004). In addition, with the global outbreak of the COVID-19 pandemic, the provision of healthcare for depression has been further challenged. To minimize the risk of infection, people have been advised to physically distance themselves (Chu et al., 2020). As a result, face-to-face consultations with healthcare professionals were substantially reduced (Reich et al., 2021).

To ensure adequate treatment while adhering to infection control measures, electronic health (e-health) services such as telemedicine have gained a more important role in the delivery of healthcare during the COVID-19 pandemic. Telemedicine includes synchronous interventions delivered via telecommunication audio-visual technology, such as video consultations (VC) and telephone consultations (TC), where both the patient and the healthcare professional are present. This allows for remote yet real-time direct interactions, ensuring access to care not only for those avoiding exposure to COVID-19, but also for those who are immobile or living in underserved and remote areas (Gude et al., 2021). Telemedicine reduces travel time, costs and stress, as well as patients' fear of stigmatization related to seeking face-to-face consultations (Pruitt et al., 2014). Numerous studies have shown the efficacy and effectiveness of telemedicine (Berryhill et al., 2019; Fernandez et al., 2021; Osenbach et al., 2013). Therefore, even before the emergence of the COVID-19 pandemic, telemedicine was considered a promising approach to narrow the treatment gap.

Prior to the pandemic, the use of telemedicine in Germany was limited. In 2019, only 3000 VC were conducted across the country (*KBV - Immer Mehr Praxen Greifen Zur Kamera - Zahl Der Videosprechstunden Auf Über Eine Million Gestiegen*, n.d.). This was largely attributed to the restrictions imposed by German healthcare insurance regulations, which limited the use and reimbursement of VC. In addition to legal policies, the uptake of telemedicine is significantly influenced by users' acceptance of the technology, which is, in turn, influenced by their attitudes (Broens et al., 2007). According to the Unified Theory of Technology Acceptance and Use (UTAUT; Venkatesh et al., 2003), which is one of the prevailing theory frameworks explaining technology acceptance (Alqudah et al., 2021), acceptance is defined as the behavioural intent to use a specific technology. Intentions are, in turn, influenced by performance expectancy, effort expectancy, social influences, and facilitating conditions. This is consistent with findings from Patel et al. (2020), who demonstrated that the acceptability of e-health among adults with depression, anxiety, and somatoform disorders varied based on associated motivation, initial individual perceptions and expectations, and the level of support offered. A recent review revealed that patients with depression who used telemedicine found it at least as acceptable as face-to-face consultations (Guaiana et al., 2021). Despite its potential benefits, the widespread adoption of telemedicine has been hindered by several barriers. These barriers include a lack of awareness and proficiency in using the technology (Cowan et al., 2019; Scott Kruse et al., 2018), as well as uncertainty regarding its efficacy, quality, and privacy. Additionally, concerns about the absence of personal contact, especially during a patient's crisis, have been raised (Cowan et al., 2019).

The COVID-19 pandemic has underscored the importance of e-health services as an essential and accepted component of the healthcare system. These services have enabled a transition to remote healthcare, and thus a certain state of preparedness for future crises. In response to the public health measures implemented during the COVID-19 pandemic, insurance regulations were relaxed in most countries, facilitating the uptake and reimbursement of telemedicine (Kinoshita et al., 2020). In Germany, healthcare insurances temporarily permitted unlimited delivery of VC and TC for medical and psychotherapeutic care (*KBV - Coronavirus: Videosprechstunden Unbegrenzt Möglich*, n.d.). As a result, healthcare professionals in Germany (Albrecht et al., 2021) and other countries (Li et al., 2021) have increasingly offered VC and TC. This was especially crucial for vulnerable individuals, including those suffering from depression, as the lockdown measures in Germany have led to reduced access to healthcare and exacerbation of depressive illnesses (Czaplicki et al., 2022). Therefore, the use of telemedicine for individuals suffering from depression may have changed during the pandemic. Additionally, attitudes towards telemedicine may have shifted after an extended period of the pandemic that increased familiarity and experience with VC and TC. As people become more familiar with a technology, their acceptance of it tends to improve (Christensen et al., 2020; Thomas et al., 2021). However, there is currently limited understanding of the changes in telemedicine usage and attitudes that may have arisen during the COVID-19 pandemic. To address this gap, our study aims to assess changes in usage behaviour and intended use of VC and TC during the pandemic, and to explore attitudes and perceived barriers among individuals with depression who have experienced a prolonged period of increased availability of remote health services. The findings of the present study will help identify areas for intervention to better meet patients' needs, overcome concerns and difficulties, and ultimately facilitate the uptake of e-health services for people affected by depression.

## 2 Objective

Based on a series of nationally representative online surveys that were conducted during the COVID-19 pandemic, the objectives of the present study were:

1) to examine whether the reported use of VC and TC and intended future use of VC with healthcare professionals among adults with diagnosed depression changed over three different timepoints during the COVID-19 pandemic;
2) to explore attitudes towards VC and TC and the main perceived barriers towards using e-health among adults with diagnosed depression at a timepoint after a prolonged time of the COVID-19 pandemic; and
3) to understand differences in results between subgroups based on sociodemographic (gender and age) and clinical characteristics (currently acute vs. residual depressive symptoms vs. symptom-free).

## 3 Methods

### 3.1 Study design

The first objective of this study concerning changes over the course of the COVID-19 pandemic was addressed using a quasi-longitudinal design. Nationally representative online surveys were conducted at three different timepoints (t) during the COVID-19 pandemic, with the first (t1: 26th June to 28th July 2020) around seven weeks after the first COVID-19 related lockdown in Germany had ended, the second (t2: 17th to 28th February 2021) two months into the second and most restrictive lockdown in Germany, and the third (t3: 16th to 28th September 2021) when COVID-19 vaccinations were broadly available and the restrictions were loosened. For the second objective a cross-sectional design was applied by solely using the survey at t3.

### 3.2 Procedures

The surveys were conducted in the context of the German Depression Barometer that is carried out annually by the German Depression Foundation. The German Depression Barometer is a nationally representative online survey exploring views on and attitudes towards depression in the general German-speaking population. In addition to the regular annual surveys (t1, t3), an additional survey (t2) was conducted in early 2021 to capture the experiences during the second lockdown in Germany. The samples of each survey were independent. The access panel pool was provided by Respondi AG, a certified market and target group research company (ISO 26362). All respondents were registered in the panel and had declared their willingness to participate in anonymous surveys. An expense allowance was made in the form of points (equivalent to 1 €), which could be used for online purchases. Each sample composition was representative of the German resident population by multi-layered quota sampling based on the interleaved characteristics of gender, age, and federal state residence in accordance with the current population estimation of the Federal Statistical Office.

### 3.3 Measures

All three surveys consisted of the same questions. Additional questions concerning attitudes towards VC and TC and barriers towards e-health were included in the last survey at t3 (see 3.3.4, 3.3.5). Pertaining to the novelty of the pandemic situation, the majority of the measures have been developed purposefully for the present work. The questions assessing use (Section 3.3.2) and intended future use of VC and TC (Section 3.3.3) have been developed by the authors (AC and UH). The questions assessing clinical characteristics (Section 3.3.1) and attitudes towards VC and TC (Section 3.3.4) have been adapted from previous assessments conducted by the German Depression Foundation. The questionnaire assessing barriers towards e-health (Section 3.3.5) has been developed for the purpose of the present manuscript (authors SG and HR). Basic sociodemographic information was assessed in the beginning of each survey.

#### 3.3.1 Clinical characteristics

To identify those suffering from depression, the survey included a self-report about previous experiences with depression. Solely respondents who chose the response option “Yes, I have already been diagnosed with depression once” were included into the subsequent parts of the survey. The current disease state was assessed by asking whether respondents were experiencing an acute depressive episode, still suffering from residual depressive symptoms, currently symptom-free, or uncertain about it.

#### 3.3.2 Use of VC and TC

The use of VC and TC was assessed by asking whether respondents had ever used VC or TC with a healthcare professional (“yes, for the first time in the current situation”, “yes, but already before”, and “no, not yet”). For the purpose of the present analysis, all respondents that chose a “yes” option were asked to specify the services they had used by choosing from the following four options (multiple answers were allowed): “VC with a psychotherapist”, “TC with a psychotherapist”, “VC with a doctor”, and “TC with a doctor”.

#### 3.3.3 Intended future use of VC

To assess intended future use of VC for different purposes of healthcare, respondents were provided with three possible purposes for using VC (“using VC for therapy sessions”, “using VC to discuss clinical findings, laboratory results or diagnostic analyses with a doctor”, and “using VC instead of a regular doctor's visit”) and were then asked to indicate their intention to use each in the future (Likert scale, 0 = “I would definitely not use it”, 1 = “I would rather not use it”, 2 = “I would maybe use it”, 3 = “I would definitely use it”).

#### 3.3.4 Attitudes towards VC and TC

Attitudes were assessed using a set of questions that have been used in the German Depression Barometer surveys before (2017, 2020, 2021) to explore attitudes towards online self-management programs. As the contents queried were regarded as relevant for VC and TC, the wording “online self-management programmes” was replaced by “VC and TC”. The questionnaire comprised the following six different statements: “VC and TC are too impersonal”, “I have concerns about data security when using VC and TC”, “VC and TC rather lead to a deterioration”, “VC and TC are a helpful support”, “VC and TC are an alternative to pharmacotherapy”, “VC and TC are an alternative to face-to-face psychotherapy”. Respondents were asked to indicate their agreement to each statement (Likert scale, 0 = “I strongly disagree”, 1 = “I disagree”, 2 = “I agree”, 3 = “I strongly agree”). The internal consistency in the present sample was moderate (Cronbach's alpha = 0.73).

#### 3.3.5 Barriers towards e-health

**Fig. 1:**
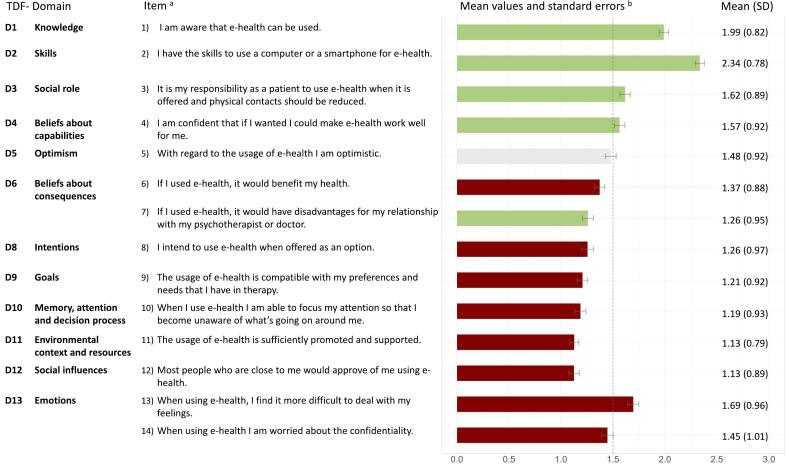
Results of the questionnaire assessing barriers to the use of e-health among adults with diagnosed depression, September 2021 (N = 1255). ^a^Statements measuring the respective TDF-domain to which respondents were asked to indicate their agreement on a Likert scale: 0 (“I strongly disagree”) to 3 (“I strongly agree”). ^b^Mean values of responses are represented by bars (red = barrier, green = facilitator, grey = neutral), the dashed line indicates the middle of the scale at a mean value of 1.50 as reference point.

Barriers were assessed using a questionnaire developed on the basis of the Theoretical Domains Framework (TDF) of behaviour change and sample items by Huijg et al. (2014). Eleven of those items were selected to serve as templates, and three separate items were additionally formulated. The present questionnaire consisted of 14 items measuring 12 TDF domains (see Fig. 1). Respondents were asked to indicate their agreement to each item (Likert scale, 0 = “I strongly disagree”, 1 = “I disagree”, 2 = “I agree”, 3 = “I strongly agree”). The internal consistency in the present sample was high (Cronbach's alpha = 0.87).

### 3.4 Statistical analyses

First, data was examined by using descriptive statistics for proportions for categorical data and central tendency and dispersion for numerical data obtained from Likert scales. Differences between the three samples in sociodemographic and clinical characteristics were analysed using Chi-squared tests. To analyse changes over time, results of the three surveys (t1–t3) were compared. Differences between the surveys and differences between subgroups based on respondents' sociodemographic and clinical characteristics were tested for statistical significance by performing logistic regressions or multivariate analyses of variance (MANOVA). For subgroup analyses, the three samples of the surveys were combined. For MANOVA, partial eta squared (η_p_^2^) was used as a measure of effect size with η_p_^2^ ≈ 0.01 a small, η_p_^2^ ≈ 0.06 a medium and η_p_^2^ ≈ 0.14 a large effect. Significant outcome measures were followed by post-hoc comparisons using univariate analyses of variance (ANOVA). Mean differences (*M*_diff_) in post-hoc pairwise comparisons were examined by Tukey's or Games-Howell test. Effect sizes of *M*_diff_ were calculated using Cohens' *d* with *d* ≈ 0.20 as a small, *d* ≈ 0.50 a medium, and *d* ≈ 0.80 a large effect (Cohen, 2013). Due to minor oversampling, analyses were weighted by gender, age, and federal state residence. Statistical tests were performed in RStudio (Version 1.4.1106) with a significance level of alpha = 0.05.

## 4 Results

### 4.1 Samples

Respondents aged 18–69 years with a professionally diagnosed depression were included in the present analyses, with a total of N = 3387 in all three surveys combined. Table 1 summarises sociodemographic information and clinical characteristics of the three samples separately. There were no significant differences in sociodemographic characteristics between the three samples. Regarding clinical characteristics, there were more respondents with acute depressive symptoms at t2 (*p* < .001).

**Table 1:** Sociodemographic and clinical characteristics of the samples at t1, t2, and t3.

Variables	Timepoint of survey			Statistics
	t1 (N = 1094)	t2 (N = 1038)	t3 (N = 1255)	
	n (%)	n (%)	n (%)	
Gender				χ^2^_2_ = 0.57, *p* = .751
Female	639 (57.00)	622 (59.92)	748 (58.95)	
Male	455 (43.00)	416 (40.08)	507 (41.05)	
Age				χ^2^_8_ = 6.97, *p* = .540
18–29 years	135 (13.16)	122 (11.75)	179 (15.40)	
30–39 years	164 (15.57)	164 (15.80)	184 (15.16)	
40–49 years	202 (18.72)	214 (20.62)	241 (19.03)	
50–59 years	357 (31.79)	318 (30.64)	372 (29.11)	
60–69 years	236 (20.76)	220 (21.19)	279 (21.29)	
Education level				χ^2^_6_ = 1.86, *p* = .932
Basic school^a^	167 (15.12)	174 (16.76)	195 (15.40)	
Secondary school^b^	474 (43.14)	451 (43.45)	540 (42.82)	
German *Abitur/Fachabitur*^c^	447 (41.19)	408 (39.31)	511 (41.05)	
No graduation level (so far)	6 (0.56)	5 (0.48)	9 (0.73)	
Disease state				**χ**^**2**^_**6**_ **=** **24.58, *p*** **<** **.001**
Acute depressive episode	197 (18.26)	260 (25.05)	236 (18.87)	
Residual depressive symptoms	442 (40.41)	422 (40.66)	509 (40.73)	
Symptom-free	417 (37.81)	328 (31.60)	464 (36.77)	
Uncertain	38 (3.52)	28 (2.70)	46 (3.63)	

Notes. t1 = June/July 2020, t2 = February 2021, t3 = September 2021, χ ^2^ = Chi square.

Statistically significant results (*p* < .05) are bold.

a Basic school: German *Hauptschule/Volksschule* with 9 years of education.

b Secondary school: German *Realschule* with 10 years of education.

c German *Abitur/ Fachabitur* with 12–13 years of education.

### 4.2 Use of video and telephone consultations

In all three surveys, one-sixth of respondents reported having ever used VC or TC with a healthcare professional (see Table 2).

**Table 2:** Self-reported use of video and telephone consultations among adults with diagnosed depression at t1, t2, and t3.

	n/N (%)	OR	95 % CI		*p*
Video or telephone consultations with a healthcare professional					
t1 (=ref.)	179/1094 (16.51)	1.00	–	–	–
t2	210/1038 (20.23)	1.23	0.98	1.53	.075
t3	230/1255 (18.47)	1.13	0.91	1.41	.265

Video consultations with a psychotherapist					
t1 (=ref.)	62/179 (34.83)	1.00	–	–	–
t2	79/210 (37.62)	1.21	0.78	1.89	.388
t3	102/230 (44.98)	**1.64**	**1.08**	**2.53**	**.023**

Telephone consultations with a psychotherapist					
t1 (=ref.)	81/179 (45.51)	1.00	–	–	–
t2	105/210 (50.00)	1.24	0.82	1.89	.307
t3	97/230 (42.36)	0.91	0.61	1.37	.660

Video consultations with a doctor					
t1 (=ref.)	21/179 (12.29)	1.00	–	–	–
t2	27/210 (12.86)	1.22	0.65	2.29	.541
t3	32/230 (14.04)	1.17	0.65	2.17	.601

Telephone consultations with a doctor					
t1 (=ref.)	51/179 (28.09)	1.00	–	–	–
t2	55/210 (26.19)	0.85	0.53	1.38	.518
t3	60/230 (25.44)	0.83	0.52	1.33	.434

Notes. Results of multiple logistic regression analyses of use with timepoint of the survey as dependent variable and “not used” as baseline reference.

t1 = June/July 2020, t2 = February 2021, t3 = September 2021, ref. = reference category of logistic regression, OR = odds ratio, CI = confidence interval, *M* = mean, *SD* = standard deviation.

Statistically significant results (*p* < .05) are bold.

#### 4.2.1 Changes over time

The overall proportion of respondents who reported ever having used VC or TC did not change significantly over time (see Table 2). However, among those who reported having used VC or TC at t3, significantly more respondents reported having ever used VC with a psychotherapist compared to t1 (OR = 1.64, *p* = .023).

#### 4.2.2 Differences based on sociodemographic characteristics

Compared to women, men were significantly more likely to have used VC with a doctor (OR = 1.82, *p* = .016). For age groups above 39 years, increasing age was associated with significantly lower reported use of VC or TC. For instance, respondents aged 60–69 years were half as likely to report having ever used VC or TC compared to those aged 30–39 years (OR = 0.45, *p* < .001). For further results see Appendix A Tables A.1 and A.2.

#### 4.2.3 Differences based on clinical characteristics

Respondents who were currently experiencing acute depressive symptoms were more than twice as likely (OR = 2.20, *p* < .001) and respondents suffering from residual depressive symptoms were 1.8 times more likely (OR = 1.79, *p* < .001) to report ever having used VC or TC compared to those who were currently symptom-free (see Appendix A Table A.3).

### 4.3 Intended future use of video consultations

Depending on the purpose of consulting a healthcare professional, respondents' intended future use of VC was moderate to high, with the highest intended future use reported for using VC to discuss clinical findings, laboratory results or diagnostic analyses (see Appendix B Table B.1).

#### 4.3.1 Changes over time

Changes over time in respondents' intended future use of VC were not statistically significant (Λ = 1.00, *F*_3, 3380_ = 0.40, *p* = .753, η_p_^2^ < 0.001). However, on a descriptive level, mean values of responses for all three purposes were higher at t2 compared to t1 and t3 (see Appendix B Table B.1). Pairwise comparisons showed that intended future use had increased significantly for the purpose of consulting a doctor to discuss clinical findings, laboratory results or diagnostic analyses (*M*_diff_ = 0.13, 95 % CI = [0.02, 0.24], *p* = .017, *d* = −0.09), but had decreased again by t3 to a level comparable to that of t1 (*p* = .023, *d* = 0.05).

#### 4.3.2 Differences based on sociodemographic characteristics

Intended future use of VC was not significantly different based on gender (MANOVA: *p* = .039, η_p_^2^ = 0.002, post-hoc separate ANOVAs: all *p* > .05). Between age groups, significant differences were found (Λ = 0.98, *F*_3,3380_ = 25.09, *p* < .001, η_p_^2^ = 0.02). For age groups above 49 years, increasing age was associated with lower intentions to use VC. For instance, respondents aged 60–69 years were significantly less likely to intend using VC for therapy sessions compared to younger respondents aged 18–29 years (*M*_diff_ = −0.37, 95 % CI = [−0.55, −0.19], *p* < .001, d = 0.35). For further results see Appendix B Table B.2.

#### 4.3.3 Differences based on clinical characteristics

Intended future use was not significantly different based on respondents' disease state (MANOVA: *p* = .013, η_p_^2^ = 0.003, post-hoc separate ANOVAs: all *p* > .05; see Appendix B Table B.3).

### 4.4 Attitudes towards video and telephone consultations

Respondents' attitudes towards VC and TC assessed at t3 were ambivalent. The majority agreed with the statement that VC and TC are too impersonal (68.70 %, n = 862) and less than half agreed with the statements that VC and TC are an alternative to pharmacotherapy (41.78 %, n = 486) or face-to-face psychotherapy (42.34 %, n = 531). However, most respondents agreed that VC and TC can provide helpful support (61.59 %, n = 772) and tended to disagree that they lead to deterioration (72.82 %, n = 916). Regarding data security, less than half of all respondents stated to be concerned about it (41.78 %, n = 524). For proportions of all four response options see Appendix C Table C.1.

#### 4.4.1 Differences based on sociodemographic characteristics

There were no significant differences in attitudes based on gender (Λ = 0.99, *F*_3, 1246_ = 1.65, *p* = .129, η_p_^2^ = 0.008) and age (MANOVA: *p* = .002, η_p_^2^ = 0.02; post-hoc tests: *p* > .05; see Appendix C Table C.2).

#### 4.4.2 Differences based on clinical characteristics

Attitudes were not significantly different based on respondents' disease state (Λ = 1.00, *F*_3, 1246_ = 0.66, *p* = .684, η_p_^2^ = 0.003; see Appendix C Table C.3).

### 4.5 Barriers to the use of e-health services

For an overview of TDF domains, corresponding items, and mean values of responses see Fig. 1, for proportions of all four response options see Appendix D Table D.1. The two most endorsed barriers towards the use of e-health were found in the domains *environmental context and resources* and *social influences*. Most respondents felt that promotion and support for e-health in Germany was insufficient and they did not believe that their significant others would approve of them using e-health. Further key barriers were found in the domains *goals*, *intentions*, and *memory, attention and decision process*. Important enablers to the use of e-health were found in *knowledge* and *skills*, implying that a vast majority of respondents were aware of e-health and were confident about their own ability to use it.

#### 4.5.1 Differences based on sociodemographic characteristics

Analyses yielded significant gender differences (Λ = 0.97, *F*_14, 1238_ = 2.08, *p* = .011, η_p_^2^ = 0.02). For women, *knowledge* (*M*_*women*_ = 2.04, *SD*_*women*_ = 0.81, *M*_diff_ = −0.12, 95 % CI = [−0.21, −0.03], *p* = .012, *d* = 0.15; see Appendix D Table D.2) was a stronger enabler than for men. Responses were also significantly different between age groups (Λ = 0.95, *F*_14, 1238_ = 4.85, *p* < .001, η_p_^2^ = 0.05). For respondents older than 49 years the domains *skills*, *social role*, *beliefs about capabilities*, *optimism*, *intentions*, *goals*, and *social influences* were perceived as stronger barriers to the use of e-health than for younger respondents (see Table 3.1).

**Table 3.1:** Barriers to the use of e-health by age, N = 1255.

TDF-domain		Item^a^	Age in years					ANOVA^a^
			18–29 (N = 179)	30–39 (N = 184)	40–49 (N = 241)	50–59 (N = 372)	60–69 (N = 279)	
			*M* (*SD*)	*M* (*SD*)	*M* (*SD*)	*M* (*SD*)	*M* (*SD*)	
D1	Knowledge	1) I am aware that e-health can be used.	2.08 (0.81)	2.07 (0.85)	1.95 (0.82)	1.95 (0.81)	1.96 (0.81)	**F** **=** **5.21, *p*** **=** **.023, η**_**p**_^**2**^ **=** **0.004**
D2	Skills	2) I have the skills to use a computer or a smartphone for e-health.	2.51 (0.75)	2.41 (0.82)	2.36 (0.77)	2.26 (0.80)	2.25 (0.76)	**F** **=** **17.43, *p*** **<** **.001, η**_**p**_^**2**^ **=** **0.01**
D3	Social/professional role	3) It is my responsibility as a patient to use e-health when it is offered, and physical contacts should be reduced.	1.76 (0.86)	1.67 (0.87)	1.65 (0.89)	1.53 (0.90)	1.58 (0.90)	**F** **=** **8.44, *p*** **=** **.004, η**_**p**_^**2**^ **=** **0.007**
D4	Beliefs about capabilities	4) I am confident that if I wanted, I could make e-health work well for me.	1.72 (0.92)	1.60 (0.93)	1.68 (0.92)	1.47 (0.92)	1.47 (0.91)	**F** **=** **12.68, *p*** **<** **.001, η**_**p**_^**2**^ **=** **0.01**
D5	Optimism	5) With regard to the usage of e-health I am optimistic.	1.65 (0.91)	1.58 (0.87)	1.56 (0.93)	1.38 (0.92)	1.37 (0.91)	**F** **=** **17.59, *p*** **<** **.001, η**_**p**_^**2**^ **=** **0.01**
D6	Beliefs about consequences	6) If I used e-health, it would benefit my health.	1.51 (0.90)	1.37 (0.88)	1.46 (0.88)	1.30 (0.86)	1.31 (0.90)	**F** **=** **7.35, *p*** **=** **.007, η**_**p**_^**2**^ **=** **0.006**
		7) If I used e-health, it would have disadvantages for my relationship with my psychotherapist or doctor.	2.61 (0.95)	2.76 (0.96)	2.78 (0.95)	2.71 (0.95)	2.81 (0.92)	F = 2.21, *p* = .137, η_p_^2^ = 0.002
D8	Intentions	8) I intend to use e-health when offered as an option.	1.48 (0.99)	1.36 (0.96)	1.31 (1.01)	1.17 (0.92)	1.11 (0.97)	**F** **=** **21.53, *p*** **<** **.001, η**_**p**_^**2**^ **=** **0.02**
D9	Goals	9) The usage of e-health is compatible with my preferences and needs that I have in therapy.	1.43 (0.93)	1.29 (0.92)	1.25 (0.96)	1.13 (0.88)	1.09 (0.93)	**F** **=** **19.26, *p*** **<** **.001, η**_**p**_^**2**^ **=** **0.02**
D10	Memory, attention, and decision processes	10) When I use e-health I am able to focus my attention so that I become unaware of what's going on around me.	1.26 (0.96)	1.22 (0.98)	1.28 (0.93)	1.13 (0.90)	1.12 (0.93)	**F** **=** **3.94, *p*** **=** **.047, η**_**p**_^**2**^ **=** **0.003**
D11	Environmental context and resources	11) The usage of e-health is sufficiently promoted and supported.	1.19 (0.90)	1.22 (0.84)	1.09 (0.77)	1.11 (0.78)	1.07 (0.71)	**F** **=** **4.32, *p*** **=** **.038, η**_**p**_^**2**^ **=** **0.003**
D12	Social influences	12) Most people who are close to me would approve of me using e-health.	1.36 (0.90)	1.28 (0.93)	1.14 (0.89)	1.06 (0.85)	0.95 (0.84)	**F** **=** **31.41, *p*** **<** **.001, η**_**p**_^**2**^ **=** **0.02**
D13	Emotions	13) When using e-health, I find it more difficult to deal with my feelings.	2.16 (0.93)	2.35 (0.97)	2.35 (0.95)	2.33 (0.97)	2.30 (0.97)	F = 1.69, *p* = .194, η_p_^2^ = 0.001
		14) When using e-health I am worried about the confidentiality.	2.49 (0.97)	2.59 (0.95)	2.61 (1.02)	2.60 (1.01)	2.45 (1.05)	F = 0.13, *p* = .721, η_p_^2^ < 0.001

Notes. Barriers were assessed on a Likert scale (0 = “strongly disagree” to 3 = “strongly agree”), *M* = mean, *SD* = standard deviation.

Statistically significant results (*p* < .05) are bold.

a Post-hoc ANOVAs if MANOVA was significant.

#### 4.5.2 Differences based on clinical characteristics

Responses were significantly different based on respondents' disease state (Λ = 0.97, *F*_14, 1238_ = 3.18, *p* < .001, η_p_^2^ = 0.03). For respondents with acute depressive symptoms, the domains *skills* (*M*_diff_ = 0.18, 95 % CI = [0.01, 0.34], *p* = .026, *d* = −0.22), *social role* (*M*_diff_ = 0.23, 95 % CI = [0.05, 0.42], *p* = .008, *d* = −0.26), *beliefs about capabilities* (*M*_diff_ = 0.28, 95 % CI = [0.09, 0.47], *p* < .001, *d* = −0.31), and *optimism* (*M*_diff_ = 0.30, 95 % CI = [0.12, 0.49], *p* < .001, *d* = −0.32) were stronger barriers than for those who were symptom-free (see Table 3.2).

**Table 3.2:** Barriers to the use of e-health by disease state, N = 1255.

TDF-domain		Item^a^	Disease state				ANOVA^a^
			Acute episode (N = 236)	Residual symptoms (N = 509)	Symptom-free (N = 464)	Uncertain (N = 46)	
			*M* (*SD*)	*M* (*SD*)	*M* (*SD*)	*M* (*SD*)	
D1	Knowledge	1) I am aware that e-health can be used.	1.90 (0.88)	2.00 (0.79)	2.03 (0.80)	1.85 (0.98)	F = 1.42, *p* = .234, η_p_^2^ = 0.001
D2	Skills	2) I have the skills to use a computer or a smartphone for e-health.	2.24 (0.86)	2.33 (0.77)	2.42 (0.74)	2.08 (0.88)	**F** **=** **3.97, *p*** **=** **.047, η**_**p**_^**2**^ **=** **0.003**
D3	Social/professional role	3) It is my responsibility as a patient to use e-health when it is offered, and physical contacts should be reduced.	1.48 (0.93)	1.61 (0.87)	1.72 (0.87)	1.39 (1.01)	**F** **=** **5.89, *p*** **=** **.015, η**_**p**_^**2**^ **=** **0.005**
D4	Beliefs about capabilities	4) I am confident that if I wanted, I could make e-health work well for me.	1.40 (0.94)	1.55 (0.92)	1.68 (0.90)	1.44 (1.01)	**F** **=** **11.16, *p*** **<** **.001, η**_**p**_^**2**^ **=** **0.009**
D5	Optimism	5) With regard to the usage of e-health I am optimistic.	1.29 (0.98)	1.49 (0.89)	1.59 (0.89)	1.31 (0.97)	**F** **=** **11.62, *p*** **<** **.001, η**_**p**_^**2**^ **=** **0.009**
D6	Beliefs about consequences	6) If I used e-health, it would benefit my health.	1.26 (0.95)	1.41 (0.87)	1.40 (0.85)	1.22 (0.95)	F = 1.70, *p* = .193, η_p_^2^ = 0.001
		7) If I used e-health, it would have disadvantages for my relationship with my psychotherapist or doctor.	1.37 (1.00)	1.27 (0.91)	1.21 (0.96)	1.11 (0.86)	**F** **=** **4.74, *p*** **=** **.030, η**_**p**_^**2**^ **=** **0.004**
D8	Intentions	8) I intend to use e-health when offered as an option.	1.19 (1.02)	1.26 (0.96)	1.30 (0.96)	1.24 (1.06)	F = 2.89, *p* = .089, η_p_^2^ = 0.002
D9	Goals	9) The usage of e-health is compatible with my preferences and needs that I have in therapy.	1.15 (0.98)	1.21 (0.93)	1.24 (0.88)	1.18 (1.02)	F = 1.76, *p* = .185, η_p_^2^ = 0.001
D10	Memory, attention, and decision processes	10) When I use e-health I am able to focus my attention so that I become unaware of what's going on around me.	1.10 (0.97)	1.17 (0.90)	1.27 (0.95)	1.07 (1.00)	**F** **=** **4.15, *p*** **=** **.042, η**_**p**_^**2**^ **=** **0.003**
D11	Environmental context and resources	11) The usage of e-health is sufficiently promoted and supported.	1.08 (0.82)	1.12 (0.78)	1.18 (0.79)	0.93 (0.74)	F = 0.97, *p* = .324, η_p_^2^ < 0.001
D12	Social influences	12) Most people who are close to me would approve of me using e-health.	1.18 (0.93)	1.17 (0.86)	1.09 (0.89)	0.90 (0.86)	F = 2.53, *p* = .112, η_p_^2^ = 0.002
D13	Emotions	13) When using e-health, I find it more difficult to deal with my feelings.	2.22 (0.99)	2.25 (0.93)	2.41 (0.98)	2.34 (0.98)	**F** **=** **6.99, *p*** **=** **.008, η**_**p**_^**2**^ **=** **0.006**
		14) When using e-health I am worried about the confidentiality.	1.56 (1.05)	1.43 (0.97)	1.40 (1.02)	1.54 (1.03)	F = 2.22, *p* = .136, η_p_^2^ = 0.002

Notes. Barriers were assessed on a Likert scale (0 = “strongly disagree” to 3 = “strongly agree”), *M* = mean, *SD* = standard deviation.

Statistically significant results (*p* < .05) are bold.

a Post-hoc ANOVAs if MANOVA was significant.

## 5 Discussion

### 5.1 Principal findings

The proportion of adults with depression who ever used VC or TC with healthcare professionals did not significantly change over time. However, among users, there was an increase in reported use of VC with psychotherapists. The intended future use of VC for healthcare varied depending on the purpose of the consultation. Significant differences over time were only found for using VC to discuss clinical findings, laboratory results and diagnostic analyses with a doctor, with higher intentions reported during the second survey conducted during lockdown in Germany. Respondents' attitudes towards VC and TC assessed after one and a half years into the pandemic were ambivalent. Key barriers to using e-health services were found to be within the *environmental context and resources* as well as within *social influences*.

At t2, respondents indicated a higher intention to use VC to consult with doctors regarding clinical findings, laboratory results and diagnostic analyses in the future compared to t1 and t3. This suggests that a patient's intention can fluctuate based on internal and external factors (Drieschner et al., 2004). Unlike the other two surveys, the t2 survey conducted in February 2021 was during a period of renewed lockdown measures to curb the spread of COVID-19, with high COVID-19 incidence rates and limited access to vaccinations against the virus. According to the UTAUT (Venkatesh et al., 2003), the construct of performance expectancy, which encompasses perceived benefits and relative advantage, is one of the most important determinants that influence the intention to use a specific technology. During the lockdown in February 2021, respondents might have perceived the benefits and advantages of VC to be greater. However, the overall effect of fluctuation in intended future use was small, and not significant for the purposes of using VC for therapy sessions and doctors' visits. Thus, patients' willingness to use these options seemed to be rather robust to external events during the time of our surveys. An explanation could lie within the constructs of social influences and facilitating conditions that influence intentions (Venkatesh et al., 2003). Both are represented in the assessment of barriers to the use of e-health within the domains *environmental context and resources* and *social influences* that were perceived as particularly hindering.

Although intended future use of VC with a doctor had increased at t2, reported use at t3 hadn't changed. This affirms that intentions are not necessarily directly translated into behaviour, a phenomenon also called the “intention-behaviour gap” (Sheeran, 2002).

In all three surveys, respondents had lower intentions to use VC for consulting a psychotherapist than for consulting a doctor. A majority of respondents indicated that they perceived VC and TC with healthcare professionals as too impersonal. This might be particularly true for psychotherapy, given the importance of a strong therapeutic relationship for its outcome (Horvath et al., 2011). Another reason for lower intentions to use VC for therapy sessions could be patients' concerns about data security and confidentiality, which may be perceived as more important in the case of mental illness due to fear of stigma. Nevertheless, the use of VC with a psychotherapist was consistently higher than with a doctor. One possible explanation may be that doctors and psychotherapists had not implemented remote consultations to the same extent. According to the results of a representative survey study in Germany (Albrecht et al., 2021), a majority of psychotherapists had started delivering psychotherapy remotely in 2020, and continued to do so at a similarly high level in 2021. In contrast, far fewer doctors had started offering remote consultations in 2020, and their offer had even declined by 2021. A therapy session, in which the conversation is of primary interest, may be easier to conduct remotely than a doctors' visit, which often requires a physical examination for diagnosis and indication (Albrecht et al., 2021). This issue could be addressed by setting clear indications for which purposes are suitable for telemedicine and which may require a face-to-face consultation (Isautier et al., 2020).

At t3, after one and a half years of the COVID-19 pandemic and the associated increased availability of remote health services, adults suffering from depression have reported mixed attitudes towards using VC and TC with healthcare professionals. While respondents mostly viewed VC and TC as helpful support, they were less likely to see them as viable alternatives to traditional face-to-face psychotherapy or pharmacotherapy. This finding reinforces previous research (Wind et al., 2020) suggesting that a blended care approach may be suitable in the post-pandemic era. Blended care involves combining face-to-face and remote consultations, such as conducting consultations for specific purposes (e.g., discussing clinical findings, laboratory results or diagnostic analyses) via telemedicine in addition to traditional face-to-face consultations. Adopting this approach would promote long-term familiarity with the technology, enabling both patients and healthcare professionals to be more flexible and adapt to remote healthcare during future crises. However, successful implementation requires addressing factors that hinder the use of e-health services. Contrary to expectations (Scott Kruse et al., 2018), patients' lack of awareness and skills were no longer among the key barriers. Instead, the greatest perceived difficulties were related to societal factors. Both institutional and private promotion and support were deemed insufficient. One aspect of this could still be restrictions by German health insurances. The regulations of facilitated and unlimited use of VC and TC were only temporary (KBV, 2022) and a revision of current health policies regarding telemedicine is needed and still pending post-pandemic. Regarding internet and smartphone-based interventions, currently only three interventions for depression have been approved for prescription and reimbursement (*DiGA-Verzeichnis*, n.d.). However, healthcare professionals may not fully support using e-health interventions (De Witte et al., 2021), as they tend to be more reluctant to using the technology than patients (Guaiana et al., 2021; Cowan et al., 2019). To increase technology acceptance among healthcare professionals, it may be helpful to establish education and training programs, ideally integrated into medical and psychotherapeutic curricula. These programs could raise awareness of the benefits of e-health technology, improve skills, familiarity and confidence with the technology, and ultimately reduce concerns (Cowan et al., 2019; De Witte et al., 2021; Di Carlo et al., 2020). Well-trained healthcare professionals may be better able to provide support by communicating information more effectively and encouraging trust in the use of e-health services. Additionally, support could be provided by loaning suitable equipment and ensuring sufficient opportunities for technical help and guidance in case of problems. The perceived lack of support from patients' significant others may be due to overall negative views and moderate attitudes towards e-health services among the general German-speaking population (Apolinário-Hagen et al., 2017, Apolinário-Hagen et al., 2018). Therefore, in addition to patients and healthcare professionals, the general public and community are an important target group for improving acceptance of e-health services. One proposal is to create and disseminate informational videos to facilitate acceptance of e-health services (Apolinário-Hagen et al., 2018).

Adults currently suffering from acute depressive symptoms were less confident in their technical skills and their capabilities to use e-health well for themselves compared to those who were symptom-free. This suggests that during acute states of depression, patients may have difficulty using e-health services, making them more easily suitable for treating residual symptoms or milder forms of depression (Kerst et al., 2020; Topooco et al., 2017). This, again, highlights the importance of maintaining guideline-based healthcare during times of crisis to ensure that those in urgent need of treatment receive appropriate care (Czaplicki et al., 2022).

### 5.2 Limitations

The main strengths of the present study were the quasi-longitudinal approach comparing the results of three representative surveys over a period of 16 months and the large, nationally representative sample sizes that resulted from our recruitment strategy. However, several limitations need to be addressed. First, the use of online surveys can cause a biased sample, as it relies on a certain familiarity with the use of the technology. Thus, individuals with low computer-literacy might be underrepresented. Furthermore, by using self-report measures only, the accuracy of information cannot be verified. Responses may be biased due to inaccurate self-assessment or inaccurate recall. By surveying independent samples within a longitudinal approach, only population trends can be described, while changes at the individual level cannot be derived. The use of self-developed assessment measures without validation restricts the comparability of results with other studies as it has been constructed specifically for the context of the COVID-19 pandemic in Germany. It may also impede the accuracy and validity of results, as not all aspects of the construct of interest (e.g., attitudes) might have been captured. The interpretation of results regarding attitudes towards VC and TC is further limited as the items don't separately ask about VC and TC and do not distinguish more specifically between the different purposes (e.g., with a doctor or psychotherapist). Analyses of changes in use and intended future use of telemedicine are limited to the time during the pandemic, as no data was collected prior to the pandemic. Finally, the results refer to people with depression living in Germany. As far as generalizability is concerned, it must be considered that the stage of digitalisation varies greatly from country to country. According to the Digital Economy and Society Index in 2021 (European Comission, 2021), Germany ranks 11th out of 27 EU Member States. In terms of e-health implementation in healthcare, Germany is considered to be rather slow compared to relatively advanced countries (Gaebel et al., 2021).

## 6 Conclusion

Despite moderately high reported intentions to use VC for psychotherapy and ambivalent attitudes towards both VC and TC among adults with depression, the reported use of VC with a psychotherapist increased during the COVID-19 pandemic. This suggests that, during times of crisis, patients' usage behaviour may be more dependent on regulations and circumstances than on their intentions and attitudes. This highlights the importance of regulatory and legislative requirements, as well as the need to ease restrictions on the use of telemedicine. In contrast, the use of VC and TC with a doctor remained consistently low during this period. Given that a lack of promotion and support for e-health services was perceived as a major barrier, urgent improvements are needed at the societal, professional, and personal levels to support the use of these services.

## Funding

The study was funded by Deutsche Bahn Stiftung gGmbH.

## Ethics statement

The Ethics Committee of the Department of Medicine at the Goethe University Frankfurt (Germany) affirmed that ethical review and approval were not required for the current study on human participants in accordance with the local legislation and institutional requirements. A written appraisal waiver was given. Written informed consent for participation was not required for this study in accordance with the national legislation and the institutional requirements.

## Declaration of competing interest

The authors declare that they have no known competing financial interests or personal relationships that could have appeared to influence the work reported in this paper. The German Depression Foundation is an independent non-profit foundation under civil law, financed primarily by donations, endowments, grants and third-party funding for projects and research. It also receives income from its business operations but works independently of the pharmaceutical industry.

## Appendix A Supplementary data

Appendices A - D with supplementary tables Image 1
