# Supplementary material for: Telemedicine during the COVID-19 pandemic in Germany: Results from three nationally representative surveys on use, attitudes and barriers among adults affected by depression
Source: Internet Interv. 2023 Apr 19;32:100622. doi: 10.1016/j.invent.2023.100622 (PMC10114311; doi:10.1016/j.invent.2023.100622)
Supplement: Supplementary file 1 — Appendices A - D with supplementary tables [file mmc1.docx]

# Appendices

## Appendix A

| **Table A.1**  Self-reported use of video and telephone consultations by gender, N=3387 | | | | | |
| --- | --- | --- | --- | --- | --- |
|  |  | | | | |
|  | n/N (%) | OR | 95% CI | | *p* |
| Video or telephone consultations with a health care professional |  |  |  |  |  |
| Female (=ref.) | 355/2009 (17.74) | *1.00* | ­– | – | ­– |
| Male | 264/1378 (19.29) | 1.08 | 0.90 | 1.29 | .425 |
|  |  |  |  |  |  |
| Video consultations with a psychotherapist |  |  |  |  |  |
| Female (=ref.) | 149/355 (42.12) | *1.00* | ­– | – | ­– |
| Male | 94/264 (36.19) | 0.73 | 0.52 | 1.04 | .084 |
| Telephone consultations with a psychotherapist |  |  |  |  |  |
| Female (=ref.) | 154/355 (43.43) | *1.00* | ­– | – | ­– |
| Male | 129/264 (49.25) | 1.30 | 0.93 | 1.81 | .126 |
| Video consultations with a doctor |  |  |  |  |  |
| Female (=ref.) | 38/355 (10.60) | *1.00* | ­– | – | ­– |
| Male | 42/264 (16.42) | **1.82** | **1.12** | **2.98** | **.016** |
| Telephone consultations with a doctor |  |  |  |  |  |
| Female (=ref.) | 97/355 (27.14) | *1.00* | ­– | – | ­– |
| Male | 69/264 (25.47) | 0.86 | 0.58 | 1.26 | .438 |
| *Notes.* Results of multiple logistic regression analyses of use with gender as dependent variable and “not used” as baseline reference  t1 = June/July 2020, t2 = February 2021, t3 = September 2021, ref. = reference category of logistic regression, OR = odds ratio, CI = confidence interval, *M* = mean, *SD* = standard deviation  Statistically significant results (*p*<.05) are **bold**. | | | | | |

| **Table A.2**  Self-reported use of video and telephone consultations by age, N=3387 | | | | | |
| --- | --- | --- | --- | --- | --- |
|  |  | | | | |
|  | n/N (%) | OR | 95% CI | | *p* |
| Video or telephone consultations with a health care professional |  |  |  |  |  |
| 18-29 years | 100/436 (22.91) | 0.83 | 0.62 | 1.12 | .232 |
| 30-39 years (=ref.) | 129/512 (25.34) | *1.00* | – | – | – |
| 40-49 years | 130/657 (19.79) | **0.72** | **0.55** | **0.95** | **.022** |
| 50-59 years | 168/1047 (16.05) | **0.56** | **0.43** | **0.72** | **<.001** |
| 60-69 years | 92/735 (12.43) | **0.45** | **0.33** | **0.61** | **<.001** |
|  |  |  |  |  |  |
| Video consultations with a psychotherapist |  |  |  |  |  |
| 18-29 years | 57/100 (56.73) | 1.17 | 0.69 | 1.98 | .570 |
| 30-39 years (=ref.) | 66/129 (51.15) | *1.00* | – | – | – |
| 40-49 years | 50/130 (38.76) | **0.58** | **0.35** | **0.95** | **.033** |
| 50-59 years | 51/168 (30.30) | **0.40** | **0.25** | **0.65** | **<.001** |
| 60-69 years | 19/92 (20.45) | **0.23** | **0.12** | **0.43** | **<.001** |
| Telephone consultations with a psychotherapist |  |  |  |  |  |
| 18-29 years | 55/100 (55.77) | 1.34 | 0.79 | 2.28 | .284 |
| 30-39 years (=ref.) | 63/129 (48.48) | *1.00* | – | – | – |
| 40-49 years | 60/130 (46.09) | 0.91 | 0.55 | 1.51 | .728 |
| 50-59 years | 75/168 (44.85) | 0.86 | 0.53 | 1.38 | .528 |
| 60-69 years | 30/92 (32.95) | 0.57 | 0.32 | 1.02 | .060 |
| Video consultations with a doctor |  |  |  |  |  |
| 18-29 years | 17/100 (17.31) | 1.52 | 0.73 | 3.17 | .262 |
| 30-39 years (=ref.) | 18/129 (14.39) | *1.00* | – | – | – |
| 40-49 years | 11/130 (8.53) | 0.56 | 0.24 | 1.25 | .164 |
| 50-59 years | 21/168 (12.73) | 0.85 | 0.43 | 1.71 | .648 |
| 60-69 years | 13/92 (14.77) | 0.88 | 0.39 | 1.94 | .758 |
| Telephone consultations with a doctor |  |  |  |  |  |
| 18-29 years | 7/100 (6.73) | **0.30** | **0.11** | **0.70** | **.009** |
| 30-39 years (=ref.) | 24/129 (18.32) | *1.00* | – | – | – |
| 40-49 years | 40/130 (31.01) | **2.01** | **1.12** | **3.64** | **.020** |
| 50-59 years | 50/168 (29.88) | **1.80** | **1.04** | **3.19** | **.040** |
| 60-69 years | 45/92 (48.86) | **4.02** | **2.19** | **7.56** | **<.001** |
| *Notes.* Results of multiple logistic regression analyses of use with age as dependent variable and “not used” as baseline reference. The age of 30-39 years was chosen as reference category to ensure that differences among young adults can become visible and differences between older adults are not artificially raised in comparison to young adults.  t1 = June/July 2020, t2 = February 2021, t3 = September 2021, ref. = reference category of logistic regression, OR = odds ratio, CI = confidence interval, *M* = mean, *SD* = standard deviation  Statistically significant results (*p*<.05) are **bold**. | | | | | |

| **Table A.3**  Self-reported use of video and telephone consultations by disease state, N=3387 | | | | | |
| --- | --- | --- | --- | --- | --- |
|  |  | | | | |
|  | n/N (%) | OR | 95% CI | | *p* |
| Video or telephone consultations with a health care professional |  |  |  |  |  |
| Acute episode | 172/693 (24.89) | **2.20** | **1.71** | **2.81** | **<.001** |
| Residual symptoms | 284/1373 (20.84) | **1.79** | **1.44** | **2.23** | **<.001** |
| Symptom-free (= ref.) | 149/1209 (12.33) | *1.00* | – | – | – |
| Uncertain | 14/112 (12.61) | 1.02 | 0.55 | 1.78 | .934 |
|  |  |  |  |  |  |
| Video consultations with a psychotherapist |  |  |  |  |  |
| Acute episode | 72/172 (42.11) | 1.26 | 0.78 | 2.05 | .342 |
| Residual symptoms | 112/284 (39.44) | 1.10 | 0.71 | 1.72 | .664 |
| Symptom-free (= ref.) | 51/149 (34.69) | *1.00* | – | – | – |
| Uncertain | 8/14 (57.14) | 2.96 | 0.91 | 10.17 | .073 |
| Telephone consultations with a psychotherapist |  |  |  |  |  |
| Acute episode | 82/172 (47.95) | **1.87** | **1.17** | **3.01** | **.009** |
| Residual symptoms | 150/284 (53.00) | **2.32** | **1.52** | **3.59** | **<.001** |
| Symptom-free (= ref.) | 46/149 (31.08) | *1.00* | – | – | – |
| Uncertain | 5/14 (35.71) | 1.00 | 0.28 | 3.17 | .995 |
| Video consultations with a doctor |  |  |  |  |  |
| Acute episode | 12/172 (6.98) | **0.23** | **0.11** | **0.45** | **<.001** |
| Residual symptoms | 33/284 (11.97) | **0.40** | **0.23** | **0.70** | **.001** |
| Symptom-free (= ref.) | 34/149 (23.13) | *1.00* | – | – | – |
| Uncertain | 1/14 (7.14) | 0.16 | 0.01 | 0.87 | .085 |
| Telephone consultations with a doctor |  |  |  |  |  |
| Acute episode | 39/172 (22.22) | 0.68 | 0.40 | 1.14 | .143 |
| Residual symptoms | 70/284 (24.38) | 0.79 | 0.50 | 1.26 | .323 |
| Symptom-free (= ref.) | 50/149 (33.11) | *1.00* | – | – | – |
| Uncertain | 7/14 (50.00) | 3.35 | 0.94 | 12.26 | .062 |
| *Notes.* Results of multiple logistic regression analyses of use with disease state as dependent variable and “not used” as baseline reference  t1 = June/July 2020, t2 = February 2021, t3 = September 2021, ref. = reference category of logistic regression, OR = odds ratio, CI = confidence interval, *M* = mean, *SD* = standard deviation  Statistically significant results (*p*<.05) are **bold**. | | | | | |

## Appendix B

| **Table B.1**  Intended future use of video consultations for different purposes of healthcare | | | | | | |
| --- | --- | --- | --- | --- | --- | --- |
| Purpose of video consultation |  | Timepoint of survey | | |  |  |
|  |  | t1  (N=1094) | t2  (N=1038) | t3  (N=1255) |  |  |
|  |  | *M* (*SD*) | *M* (*SD*) | *M* (*SD*) |  |  |
|  |  |  |  |  |  |  |
| For therapy sessions |  | 1.26 (1.08) | 1.35 (1.04) | 1.30 (1.10) |  |  |
|  |  |  |  |  |  |  |
| To discuss clinical findings, laboratory results or diagnostic analyses with a doctor |  | 1.59 (1.10) | 1.72 (1.07) | 1.60 (1.11) |  |  |
|  |  |  |  |  |  |  |
| Instead of a regular doctors’ visit |  | 1.45 (1.09) | 1.55 (1.07) | 1.46 (1.11) |  |  |
| *Notes.* Intended future use was assessed on a Likert scale (0 = “definitely not” to 3 = “definitely yes”).  t1 = June/July 2020, t2 = February 2021, t3 = September 2021, *M* = mean, *SD* = standard deviation  MANOVA was not significant, thus no post-hoc ANOVAs were conducted. | | | | | | |

| **Table B.2**  Intended future use of video consultations for different purposes of healthcare by sociodemographic characteristics, N=3387 | | | | | | | | | | | |
| --- | --- | --- | --- | --- | --- | --- | --- | --- | --- | --- | --- |
| Purpose of video consultation |  | Gender | |  |  | Age in years | | | | |  |
|  |  | Female  (N=2009) | Male  (N=1378) |  |  | 18-29 (N=436) | 30-39 (N=512) | 40-49 (N=657) | 50-59 (N=1047) | 60-69 (N=735) |  |
|  |  | *M* (*SD*) | *M* (*SD*) | ANOVA ^a^ |  | *M* (*SD*) | *M* (*SD*) | *M* (*SD*) | *M* (*SD*) | *M* (*SD*) | ANOVA ^a^ |
|  |  |  |  |  |  |  |  |  |  |  |  |
| For therapy sessions |  | 1.28 (1.08) | 1.34 (1.07) | **–** |  | 1.45 (1.08) | 1.51 (1.09) | 1.42 (1.09) | 1.22 (1.04) | 1.07 (1.06) | **F=62.55,**  ***p*<.001, η_p_²=0.02** |
|  |  |  |  |  |  |  |  |  |  |  |  |
| To discuss clinical findings, laboratory results or diagnostic analyses with a doctor |  | 1.63 (1.10) | 1.64 (1.09) | – |  | 1.66 (1.10) | 1.74 (1.06) | 1.74 (1.11) | 1.59 (1.07) | 1.52 (1.14) | **F=11.80,**  ***p*<.001, η_p_²=0.003** |
|  |  |  |  |  |  |  |  |  |  |  |  |
| Instead of a regular doctors’ visit |  | 1.46 (1.10) | 1.53 (1.08) | **–** |  | 1.45 (1.08) | 1.67 (1.07) | 1.61 (1.09) | 1.41 (1.07) | 1.37 (1.13) | **F=12.93,**  ***p*<.001, η_p_²=0.004** |
| *Notes.* Intended future use was assessed on a Likert scale (0 = “definitely not” to 3 = “definitely yes”). *M* = mean, *SD* = standard deviation  ^a^ post-hoc ANOVAs if MANOVA was significant.  Statistically significant results (*p*<.05) are **bold**. | | | | | | | | | | | |

| **Table B.3**  Intended future use of video consultations for different purposes of healthcare by disease state, N=3387 | | | | | |
| --- | --- | --- | --- | --- | --- |
| Purpose of video consultation |  | Disease state | | | |
|  |  | Acute episode (N=693) | Residual symptoms (N=1373) | Symptom-free (N=1209) | Uncertain  (N=112) |
|  |  | *M* (*SD*) | *M* (*SD*) | *M* (*SD*) | *M* (*SD*) |
|  |  |  |  |  |  |
| For therapy sessions |  | 1.33 (1.10) | 1.33 (1.07) | 1.27 (1.07) | 1.04 (1.06) |
|  |  |  |  |  |  |
| To discuss clinical findings, laboratory results or diagnostic analyses with a doctor |  | 1.58 (1.14) | 1.65 (1.08) | 1.67 (1.09) | 1.35 (1.04) |
|  |  |  |  |  |  |
| Instead of a regular doctors’ visit |  | 1.46 (1.12) | 1.49 (1.09) | 1.51 (1.09) | 1.31 (1.02) |
| *Notes.* Intended future use was assessed on a Likert scale (0 = “definitely not” to 3 = “definitely yes”). *M* = mean, *SD* = standard deviation | | | | | |

## Appendix C

| **Table C.1**  Attitudes towards video and telephone consultations, N=1255 | | | | | |
| --- | --- | --- | --- | --- | --- |
| Statement |  | Response options | | | |
|  |  | “I strongly disagree” | “I disagree” | “I agree” | “I strongly agree” |
|  |  | n (%) | n (%) | n (%) | n (%) |
|  |  |  |  |  |  |
| Too impersonal |  | 155 (12.25) | 238 (19.10) | 367 (29.49) | 495 (39.16) |
|  |  |  |  |  |  |
| Concerns about data security |  | 325 (25.89) | 406 (32.34) | 294 (23.47) | 230 (18.31) |
|  |  |  |  |  |  |
| Lead to deterioration |  | 310 (24.60) | 606 (48.23) | 256 (20.48) | 83 (6.69) |
|  |  |  |  |  |  |
| Helpful support |  | 159 (12.66) | 324 (25.73) | 547 (43.71) | 225 (17.90) |
|  |  |  |  |  |  |
| Alternative to pharmacotherapy |  | 304 (24.13) | 465 (37.21) | 374 (29.70) | 112 (8.96) |
|  |  |  |  |  |  |
| Alternative to psychotherapy |  | 298 (23.61) | 426 (34.00) | 395 (31.59) | 136 (10.80) |
|  | | | | | |

| **Table C.2**  Attitudes towards video and telephone consultations by sociodemographic characteristics, N=1255 | | | | | | | | | | |
| --- | --- | --- | --- | --- | --- | --- | --- | --- | --- | --- |
| Statement |  | Gender | |  | Age in years | | | | | |
|  |  | Female  (N=748) | Male (N=507) |  | 18-29  (N=179) | 30-39 (N=184) | 40-49 (N=241) | 50-59 (N=372) | 60-69 (N=279) | |
|  |  | *M* (*SD*) | *M* (*SD*) |  | *M* (*SD*) | *M* (*SD*) | *M* (*SD*) | *M* (*SD*) | *M* (*SD*) | |
|  |  |  |  |  |  |  |  |  |  | |
| Too impersonal |  | 2.00 (1.02) | 1.90 (1.05) |  | 2.01 (0.91) | 2.03 (0.97) | 1.80 (1.09) | 1.94 (1.06) | 2.03 (1.07) | |
|  |  |  |  |  |  |  |  |  |  | |
| Concerns about data security |  | 1.36 (1.05) | 1.31 (1.06) |  | 1.38 (1.06) | 1.39 (1.00) | 1.24 (1.05) | 1.28 (1.06) | 1.45 (1.07) | |
|  |  |  |  |  |  |  |  |  |  | |
| Lead to deterioration |  | 1.08 (0.84) | 1.11 (0.85) |  | 1.22 (0.93) | 1.12 (0.86) | 1.08 (0.84) | 1.07 (0.80) | 1.03 (0.81) | |
|  |  |  |  |  |  |  |  |  |  | |
| Helpful support |  | 1.69 (0.89) | 1.63 (0.94) |  | 1.78 (0.90) | 1.63 (0.92) | 1.73 (0.97) | 1.64 (0.87) | 1.60 (0.92) | |
|  |  |  |  |  |  |  |  |  |  | |
| Alternative to pharmacotherapy |  | 1.22 (0.91) | 1.26 (0.92) |  | 1.16 (0.94) | 1.26 (0.96) | 1.23 (0.96) | 1.27 (0.88) | 1.23 (0.88) | |
|  |  |  |  |  |  |  |  |  |  | |
| Alternative to psychotherapy |  | 1.30 (0.94) | 1.28 (0.95) |  | 1.29 (0.90) | 1.34 (0.89) | 1.41 (1.01) | 1.27 (0.94) | 1.20 (0.95) | |
| *Notes.* Attitudes were assessed on a Likert scale (0 = “strongly disagree” to 3 = “strongly agree”), *M* = mean, *SD* = standard deviation  MANOVAs were not significant, thus no post-hoc ANOVAs were conducted. | | | | | | | | | |  |

| **Table C.3**  Attitudes towards video and telephone consultations by disease state, N=1255 | | | | | |
| --- | --- | --- | --- | --- | --- |
| Statement |  | Disease state | | | |
|  |  | Acute episode (N=236) | Residual  Symptoms (N=509) | Symptom-free (N=464) | Uncertain  (N=46) |
|  |  | *M* (*SD*) | *M* (*SD*) | *M* (*SD*) | *M* (*SD*) |
|  |  |  |  |  |  |
| Too impersonal |  | 1.89 (1.08) | 2.00 (0.98) | 1.97 (1.05) | 1.67 (1.19) |
|  |  |  |  |  |  |
| Concerns about data security |  | 1.44 (1.11) | 1.33 (1.04) | 1.30 (1.04) | 1.47 (1.10) |
|  |  |  |  |  |  |
| Lead to deterioration |  | 1.19 (0.93) | 1.09 (0.79) | 1.04 (0.83) | 1.17 (0.95) |
|  |  |  |  |  |  |
| Helpful support |  | 1.63 (0.96) | 1.69 (0.88) | 1.67 (0.92) | 1.55 (0.98) |
|  |  |  |  |  |  |
| Alternative to pharmacotherapy |  | 1.17 (0.95) | 1.26 (0.92) | 1.27 (0.91) | 0.99 (0.81) |
|  |  |  |  |  |  |
| Alternative to psychotherapy |  | 1.29 (1.00) | 1.31 (0.92) | 1.29 (0.95) | 1.19 (0.96) |
| *Notes.* Attitudes were assessed on a Likert scale (0 = “strongly disagree” to 3 = “strongly agree”), *M* = mean, *SD* = standard deviation  MANOVA was not significant, thus no post-hoc ANOVAs were conducted. | | | | | |

## Appendix D

| **Table D.1**  Barriers to the use of e-health, N=1255 | | | | | | | |
| --- | --- | --- | --- | --- | --- | --- | --- |
| TDF-Domain | | Item ^a^ |  | Response options | | | |
|  | |  |  | “I strongly disagree” | “I disagree” | “I agree” | “I strongly agree” |
|  | |  |  | n (%) | n (%) | n (%) | n (%) |
| D1 | Knowledge | 1. I am aware that e-health can be used. |  | 74 (5.89) | 205 (16.37) | 637 (50.73) | 339 (27.02) |
| D2 | Skills | 1. I have the skills to use a computer or a smartphone for e-health. |  | 50 (4.03) | 95 (7.50) | 495 (39.19) | 615 (49.27) |
| D3 | Social/professional role | 1. It is my responsibility as a patient to use e-health when it is offered, and physical contacts should be reduced. |  | 158 (12.50) | 357 (28.47) | 549 (43.71) | 191 (15.32) |
| D4 | Beliefs about capabilities | 1. I am confident that if I wanted, I could make e-health work well for me. |  | 169 (13.39) | 423 (33.63) | 450 (35.97) | 213 (17.02) |
| D5 | Optimism | 1. With regard to the usage of e-health I am optimistic. |  | 195 (15.40) | 442 (35.08) | 440 (35.32) | 178 (14.19) |
| D6 | Beliefs about consequences | 1. If I used e-health, it would benefit my health. |  | 217 (17.18) | 482 (38.31) | 428 (34.35) | 128 (10.16) |
|  |  | 1. If I used e-health, it would have disadvantages for my relationship with my psychotherapist or doctor. |  | 300 (23.79) | 470 (37.50) | 345 (27.50) | 140 (11.21) |
| D8 | Intentions | 1. I intend to use e-health when offered as an option. |  | 329 (25.95) | 420 (33.44) | 362 (29.01) | 144 (11.60) |
| D9 | Goals | 1. The usage of e-health is compatible with my preferences and needs that I have in therapy. |  | 325 (25.65) | 453 (36.13) | 369 (29.52) | 108 (8.71) |
| D10 | Memory, attention and decision processes | 1. When I use e-health I am able to focus my attention so that I become unaware of what’s going on around me. |  | 332 (26.35) | 475 (37.79) | 329 (26.27) | 119 (9.59) |
| D11 | Environmental context and resources | 1. The usage of e-health is sufficiently promoted and supported. |  | 262 (20.97) | 631 (50.08) | 302 (24.11) | 60 (4.84) |
| D12 | Social influences | 1. Most people who are close to me would approve of me using e-health. |  | 349 (27.56) | 474 (37.71) | 357 (28.69) | 75 (6.04) |
| D13 | Emotions | 1. When using e-health, I find it more difficult to deal with my feelings. |  | 160 (12.81) | 349 (27.72) | 460 (36.74) | 286 (22.72) |
|  |  | 1. When using e-health I am worried about the confidentiality. |  | 264 (20.95) | 386 (30.78) | 386 (30.94) | 219 (17.32) |
|  | | | | | | | |

| **Table D.2**  Barriers to the use of e-health by gender, N=1255 | | | | | | |
| --- | --- | --- | --- | --- | --- | --- |
| TDF-Domain | | Item ^a^ |  | Gender | |  |
|  | |  |  | Female  (N=748) | Male  (N=507) |  |
|  | |  |  | *M* (*SD*) | *M* (*SD*) | ANOVA ^a^ |
| D1 | Knowledge | 1. I am aware that e-health can be used. |  | 2.04 (0.81) | 1.92 (0.83) | **F=6.44,**  ***p=*.011, η_p_²=0.005** |
| D2 | Skills | 1. I have the skills to use a computer or a smartphone for e-health. |  | 2.33 (0.77) | 2.34 (0.81) | F=0.03,  *p*.870, η_p_²<0.001 |
| D3 | Social/professional role | 1. It is my responsibility as a patient to use e-health when it is offered, and physical contacts should be reduced. |  | 1.66 (0.90) | 1.56 (0.88) | F=3.50,  *p*=.062, η_p_²=0.003 |
| D4 | Beliefs about capabilities | 1. I am confident that if I wanted, I could make e-health work well for me. |  | 1.60 (0.93) | 1.52 (0.91) | F=1.81,  *p*=.179, η_p_²=0.001 |
| D5 | Optimism | 1. With regard to the usage of e-health I am optimistic. |  | 1.51 (0.92) | 1.44 (0.91) | F=2.09,  *p*=.148, η_p_²=0.002 |
| D6 | Beliefs about consequences | 1. If I used e-health, it would benefit my health. |  | 1.37 (0.90) | 1.38 (0.87) | F=0.12,  *p=*.731, η_p_²<0.001 |
|  |  | 1. If I used e-health, it would have disadvantages for my relationship with my psychotherapist or doctor. |  | 1.23 (0.94) | 1.30 (0.95) | F=1.81,  *p*=.179, η_p_²=0.001 |
| D8 | Intentions | 1. I intend to use e-health when offered as an option. |  | 1.25 (0.97) | 1.28 (0.97) | F=0.33,  *p*=.569, η_p_²<0.001 |
| D9 | Goals | 1. The usage of e-health is compatible with my preferences and needs that I have in therapy. |  | 1.21 (0.92) | 1.21 (0.93) | F=0.001,  *p*=.975, η_p_²<0.001 |
| D10 | Memory, attention, and decision processes | 1. When I use e-health I am able to focus my attention so that I become unaware of what’s going on around me. |  | 1.18 (0.94) | 1.21 (0.93) | F=0.30,  *p*=.582, η_p_²<0.001 |
| D11 | Environmental context and resources | 1. The usage of e-health is sufficiently promoted and supported. |  | 1.15 (0.78) | 1.10 (0.81) | F=0.83,  *p*=.362, η_p_²<0.001 |
| D12 | Social influences | 1. Most people who are close to me would approve of me using e-health. |  | 1.11 (0.88) | 1.16 (0.89) | F=1.01,  *p*=.315, η_p_²<0.001 |
| D13 | Emotions | 1. When using e-health, I find it more difficult to deal with my feelings. |  | 1.72 (0.95) | 1.66 (0.98) | F=1.00,  *p*=.317, η_p_²<0.001 |
|  |  | 1. When using e-health I am worried about the confidentiality. |  | 1.45 (1.01) | 1.44 (1.00) | F=0.07,  *p*=.792, η_p_²<0.001 |
| *Notes.* Barriers were assessed on a Likert scale (0 = “strongly disagree” to 3 = “strongly agree”), *M* = mean, *SD* = standard deviation  ^a^ post-hoc ANOVAs if MANOVA was significant.  Statistically significant results (*p*<.05) are **bold**. | | | | | | |
